# Preoperative identification and multidisciplinary management of von Willebrand disease in atrial septal defect closure: a case report

**DOI:** 10.3389/fcvm.2026.1835076

**Published:** 2026-07-23

**Authors:** Wan Peng, Qimei Wei, Jun Yang, Xiaohui Liu

**Affiliations:** 1 Department of Clinical Laboratory, Wuhan Asia Heart Hospital, Wuhan, China; 2 Department of Blood Transfusion, Wuhan Asia Heart Hospital, Wuhan, China

**Keywords:** atrial septal defect, case report, interventional closure, preoperative screening, von Willebrand disease, von Willebrand factor (VWF)

## Abstract

Von Willebrand disease (VWD) is an inherited bleeding disorder caused by a quantitative or functional deficiency of von Willebrand factor (VWF). We report a 31-year-old male with an atrial septal defect (ASD) whose preoperative coagulation screening revealed mildly prolonged activated partial thromboplastin time (APTT) and reduced factor VIII (FVIII) activity. Further assays confirmed decreased VWF levels, and a detailed bleeding history revealed a significant prior tendency to bleed. A multidisciplinary team (MDT) developed an individualized management plan, including preprocedural cryoprecipitate infusion. The patient subsequently underwent successful ASD closure via interventional occlusion without hemorrhagic complications. This case illustrates that even mildly prolonged APTT in surgical candidates may indicate VWD. A careful bleeding history and targeted laboratory evaluation are essential to avoid missed diagnoses, while individualized perioperative management helps ensure procedural safety. While transcatheter ASD closure is a routine procedure, its occurrence in a patient with newly diagnosed VWD and markedly reduced VWF levels is rarely reported. Transcatheter structural interventions require intraoperative heparinization; therefore, dismissing a mildly prolonged preoperative APTT as clinically insignificant could expose an undiagnosed patient with a bleeding disorder to serious hemorrhagic risk. This case adds new knowledge by illustrating a reproducible diagnostic workflow within a non-hematological cardiac center to avert severe bleeding complications.

## Introduction

Von Willebrand disease (VWD) is the most common inherited bleeding disorder, with an estimated prevalence of 0.1% to 1% in the general population (1). The disease is primarily caused by qualitative or quantitative abnormalities in von Willebrand factor (VWF) and shows diverse inheritance patterns, including both autosomal dominant and recessive forms (1, 2). VWF plays a critical role in primary hemostasis by mediating platelet adhesion to damaged vascular endothelium and acts as a carrier protein for coagulation factor VIII (FVIII), stabilizing its plasma levels and prolonging its half-life (1, 3). Consequently, VWF dysfunction can impair both platelet adhesion and the intrinsic coagulation pathway, leading to a clinical bleeding tendency.

The clinical presentation of VWD is highly heterogeneous. Mild cases may present only with minor mucocutaneous bleeding, while severe cases can experience significant postoperative hemorrhage, or even joint and deep tissue bleeding (1). Based on the type and degree of VWF deficiency, VWD is classified into type 1 (partial quantitative deficiency), type 2 (qualitative abnormality), and type 3 (complete deficiency) (2). Type 1 is the most common, but its mild symptoms are often overlooked, leading to a high risk of missed diagnosis in non-hematology settings, particularly among patients scheduled for surgery (1, 4). Preoperative coagulation screening is a key step in identifying potential bleeding disorders. For VWD patients undergoing surgery, thorough preoperative diagnosis, risk assessment, and multidisciplinary intervention are essential to avoid perioperative bleeding complications and ensure surgical safety.

This article reports the case of a patient scheduled for interventional ASD closure who was incidentally diagnosed with VWD preoperatively. Through multidisciplinary collaboration, an individualized management strategy was formulated, leading to successful surgery. This case aims to reinforce clinicians’ awareness of the importance of perioperative VWD management and provide a reference for similar clinical scenarios.

## Case presentation

**Figure 1:**
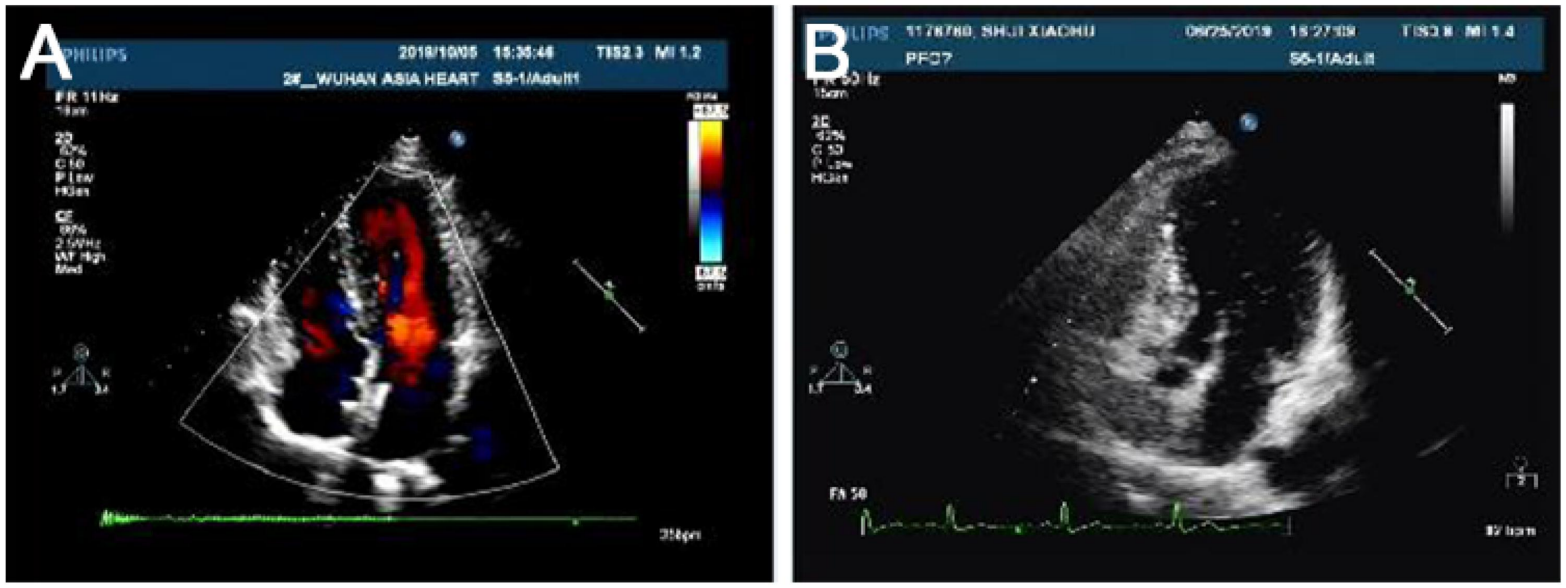
**(A)** Patient's admission echocardiogram; **(B)** patient's follow-up echocardiogram one year later.

A 31-year-old male presented to our outpatient clinic with a 5-month history of chest tightness, palpitations, and facial numbness upon exertion, which was relieved by rest. Echocardiography revealed: dense microbubble echoes in the right heart and 3–5 microbubbles in the left heart. The number of left heart microbubbles increased to approximately 30 during the Valsalva maneuver, suggesting a moderate right-to-left shunt at the atrial level (Figure 1A). He was admitted with a provisional diagnosis of “atrial septal defect”. His past medical history was unremarkable, with no history of hypertension, diabetes, infectious diseases, prior surgery/trauma, or blood transfusions. He reported no known drug allergies or family history of genetic disorders.

Physical examination revealed no abnormal bleeding signs. Routine laboratory tests showed normal complete blood count and liver/renal function. Coagulation tests indicated a mildly prolonged activated partial thromboplastin time (APTT) and decreased factor VIII activity (specific results: PT 12.0 s, APTT 43.5 s, TT 15.0 s, Fib 2.07 g/L, AT-III 99.0%, FVIII 15.9%). Results were consistent upon repeat testing, and the patient was not on anticoagulants. An APTT mixing study was performed (Table 1), suggesting a coagulation factor deficiency.

**Table 1:** Patient's APTT mixing study results.

Parameter	0 h (Immediate)	2 h (Incubated)
Patient APTT(s)	43.5	48.5
Normal Pooled Plasma APTT(s)	31.5	37.6
1:1 Mix APTT (s)	34.1	40.7
Rosner Index	5.97	6.39

A Rosner Index < 11 indicates correction; > 11 indicates no correction. These results suggest a factor deficiency.

**Figure 2:**
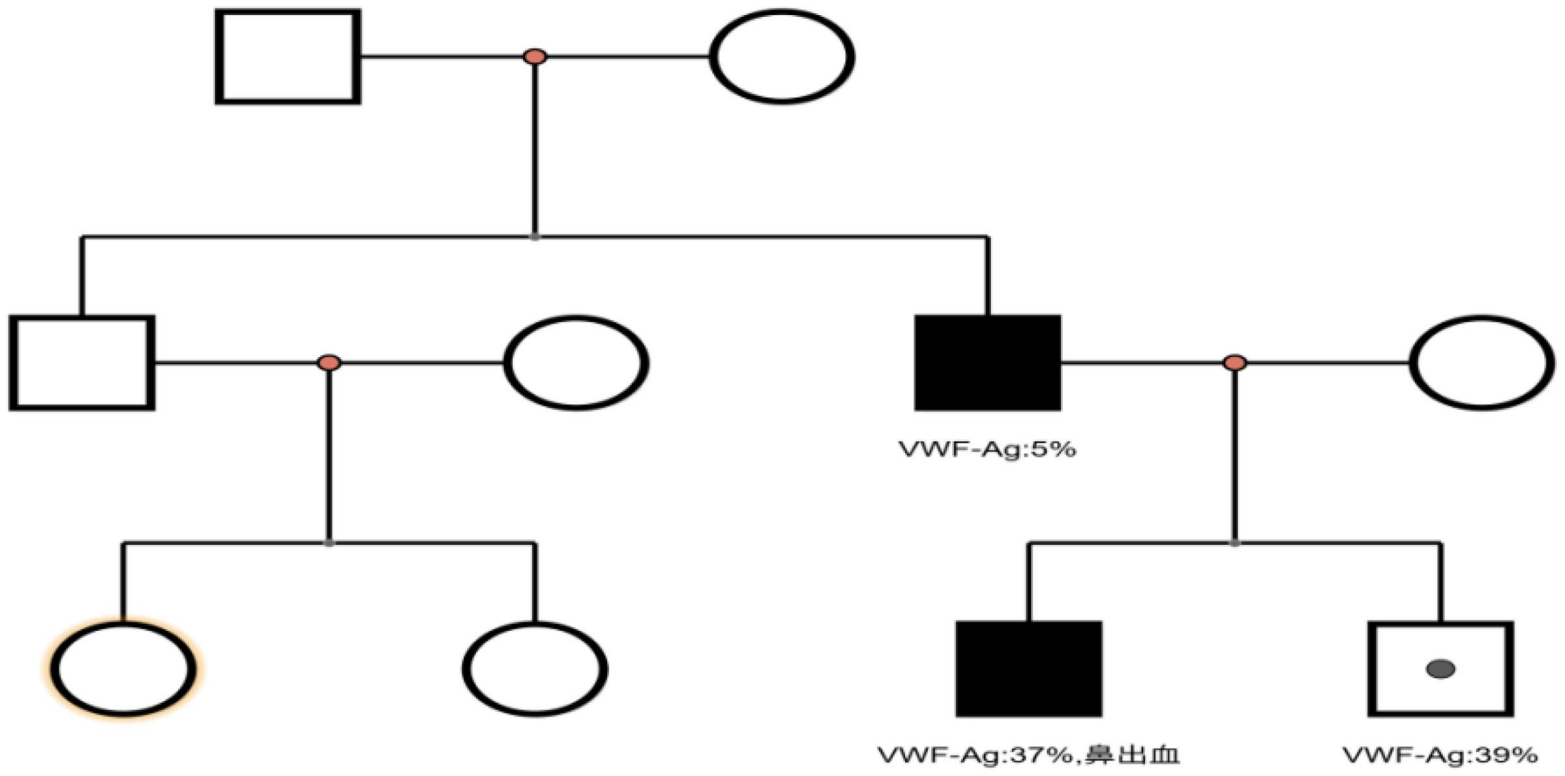
The family pedigree.

To identify the etiology, further tests were conducted: The autoimmune antibody panel was negative; other intrinsic coagulation factor activities were normal (Table 2); von Willebrand factor antigen (VWF:Ag) was 5% and VWF activity (VWF:RCo) was 2.9% (Table 3). The combination of markedly reduced VWF levels and low FVIII activity raised strong suspicion for von Willebrand disease (VWD). Further detailed history taking revealed that the patient had experienced recurrent spontaneous epistaxis during adolescence, severe enough to cause hypovolemic shock on occasion, and had undergone nasal laser surgery with poor effect. He also reported a history of gingival bleeding and bruising after venipuncture. VWF:Ag testing was performed for his two sons, with results of 39% and 37%, respectively. The elder son also had a history of spontaneous epistaxis. They were then referred to a specialized pediatric hematology clinic for formal comprehensive evaluation and long-term follow-up. At that time, no other distant relatives were available or consented to participate in coagulation testing. The family pedigree is shown in Figure 2. Integrating laboratory findings with the clinical presentation, a diagnosis of VWD was confirmed. While a formal bleeding score was not prospectively calculated during admission, we have now retrospectively evaluated the patient's bleeding history using the standardized ISTH-BAT framework. The patient scored a total of 5 points (3 points for recurrent severe epistaxis requiring medical intervention/laser surgery, 1 point for cutaneous bruising/hematoma after venipuncture, and 1 point for gingival bleeding). A score ≥4 is considered abnormal for adult males, thereby objectively confirming a significant bleeding phenotype (5).

**Table 2:** Patient's other coagulation factor activity results.

Factor	Ⅱ	Ⅴ	Ⅶ	Ⅷ	Ⅸ	Ⅹ	XI	XII
Activity (%)	100	85	108	15.9	146	92	87	102
Normal Range (%)	71–110	66–131	80–154	51.2–164	60–150	58–113	60–139	50–140

**Table 3:** Patient's VWF antigen and activity results (patient blood type O).

Parameter	VWF Antigen (VWF:Ag)	VWF Activity (VWF: RCo)	RCo/Ag ratio
Result (%)	5	2.9	0.58
Normal Reference (%)	42.0–140.8 (Type O) 66.1–176.3 (Non-Type O)	50–200	>0.7

The patient required ASD closure. After multidisciplinary discussion, interventional closure was deemed to carry a lower bleeding risk compared to open-heart surgery and was considered the preferred approach. To prevent intraoperative bleeding, 10 units of cryoprecipitate were infused 2 h preoperatively. Post-infusion monitoring showed VWF:Ag increased to 48.0%. The interventional closure procedure was then successfully performed. No significant bleeding occurred during or after the procedure. The details of the patient's medical procedure are as follows.

Periprocedural Anticoagulation: During the transcatheter procedure, standard intraoperative anticoagulation was maintained using unfractionated heparin (70 U/kg) after successful sheath insertion.

Estimated Blood Loss & Access-Site: The estimated blood loss during the procedure was minimal (<10 mL). The right femoral venous access site was managed with manual compression, and no hematoma, ecchymosis, or pseudoaneurysm occurred.

Post-procedure Antiplatelet Therapy: Following successful ASD device deployment, the patient was started on daily oral aspirin (100 mg/day) for 6 months, per standard protocol for structural interventions.

Postoperative Monitoring: Post-infusion VWF levels were monitored, and coagulation status remained stable without any clinical signs of delayed mucocutaneous or access-site bleeding during the 3-day hospitalization.

**Figure 3:**
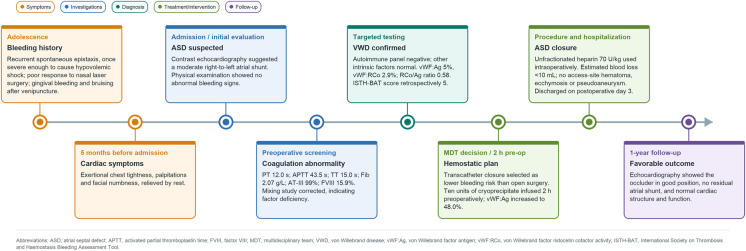
The timeline of diagnosis and treatment for this patient, from admission to the one-year follow-up.

The patient was discharged on postoperative day 3. One-year follow-up echocardiography showed the occluder in good position, with no residual atrial shunt, and normal cardiac structure and function (Figure 1B). The timeline of diagnosis and treatment for this patient, from admission to the one-year follow-up, is shown in Figure 3.

## Discussion

VWD is the most common inherited bleeding disorder. However, due to its highly heterogeneous clinical presentation and diagnostic complexity, there is a significant risk of missed diagnosis in non-hematology settings, particularly among patients scheduled for surgery (1, 4). This case report details the diagnostic evaluation and perioperative management of a patient admitted for ASD who was incidentally diagnosed with VWD before surgery, and aims to raise clinicians’ awareness of the importance of perioperative VWD management.

The diagnosis in this case began with an incidentally abnormal coagulation screening result. The patient had no obvious bleeding complaints, and the initial routine tests only revealed a mildly prolonged APTT. Such subtle abnormalities are easily overlooked in clinical practice; if the testing workflow does not include coagulation factor activity analysis, it could lead directly to a missed diagnosis. APTT prolongation suggests an abnormality in the intrinsic coagulation pathway, with primary differential diagnoses including coagulation factor deficiencies and the presence of inhibitors (e.g., lupus anticoagulant) (6). The APTT mixing study is a key initial step for differentiation. In this case, correction in both the immediate and incubated mixing tests supported a coagulation factor deficiency rather than an inhibitor. A time-dependent factor VIII inhibitor would typically show loss of correction after incubation, whereas lupus anticoagulant usually causes incomplete correction in the immediate mixing test (6). This result directed the diagnostic focus towards factor deficiency, guiding subsequent coagulation factor activity analysis, which revealed significantly reduced factor VIII (FVIII) activity (15.9%). This was the crucial step that triggered the subsequent VWD diagnostic workup.

Reduced FVIII typically first suggests hemophilia A, but VWD is an important differential diagnosis that must not be overlooked (7). VWF, as the carrier protein for FVIII, is vital for maintaining FVIII stability (1, 3). Therefore, qualitative or quantitative abnormalities of VWF can lead to secondary reductions in FVIII levels (3, 8). In Hemophilia A, FVIII activity is selectively decreased, whereas VWF antigen (VWF:Ag) and activity (VWF:RCo) remain entirely within normal ranges (7). In our patient, the parallel and marked reductions in VWF:Ag (5%), VWF:RCo (2.9%), and FVIII (15.9%) clearly directed the diagnosis toward VWD. Furthermore, acquired von Willebrand syndrome was considered less likely because of the patient's long-standing bleeding history dating back to adolescence, the absence of recognized late-onset predisposing conditions, and reduced VWF:Ag levels in both sons; however, definitive VWD subtyping and genetic confirmation were not available. The diagnostic process in our case followed this logic. After excluding other factor deficiencies and acquired causes, targeted testing for VWF antigen (VWF:Ag) and activity (VWF:RCo) was performed, confirming significantly low levels and providing laboratory confirmation for VWD. The calculated VWF:RCo/VWF:Ag ratio in this patient is approximately 0.58 (2.9%/5%). Although a ratio <0.6–0.7 typically points toward a qualitative defect (Type 2 VWD), precise interpretation of this ratio becomes highly challenging and less reliable when absolute VWF levels are profoundly low (≤5%) (1). The reduced VWF:Ag levels in both sons support, but do not confirm, a possible inherited pattern. The clinical and laboratory findings support severe type 1 or type 2 VWD, whereas definitive subtyping would require additional testing. VWF multimer analysis and genetic testing were unavailable because of local resource constraints.

Notably, the initial admission history did not reveal a significant bleeding tendency. This underscores the central importance of proactive and detailed bleeding history inquiry in diagnosing VWD (1, 9). Through targeted questioning, key historical information was obtained, including severe spontaneous epistaxis during adolescence. Family history investigation also showed reduced VWF levels and a bleeding tendency in his sons, supporting a possible inherited pattern.

Following the VWD diagnosis, the core clinical challenge was how to safely perform the cardiac defect repair. Multidisciplinary team (MDT) assessment concluded that transcatheter interventional closure, with its minimal trauma and lower bleeding risk, offered significant advantages over traditional open-heart surgery and was the preferred choice for this patient. Although the interventional procedure carried a lower risk, proactive perioperative management remained key to ensuring safety. A replacement therapy-based plan was formulated, involving the infusion of cryoprecipitate 2 h preoperatively, aiming to increase VWF levels before the procedure to reduce intraoperative bleeding risk (1, 10). Desmopressin (DDAVP) is also a treatment option for VWD. The reason it was not chosen is that its effect depends on the release of endogenous VWF stores, and in patients with severe baseline quantitative VWF deficiency (VWF:Ag ≤ 5%), such release is often insufficient or extremely unpredictable. Furthermore, DDAVP carries risks of hyponatremia and fluid retention, which warrant cautious use in patients with structural heart disease such as an atrial septal defect (11). In addition, unfortunately, at the time of surgery, our hospital pharmacy formulary did not include pure VWF concentrates or plasma-derived VWF/FVIII concentrates (e.g., Haemate P). Therefore, we chose cryoprecipitate as an efficient, rapid, and readily available source of VWF and FVIII to ensure immediate and reliable hemostasis during the perioperative period. In this case, infusion of 10 units of cryoprecipitate raised VWF:Ag to 48.0%. The multidisciplinary team then proceeded with the minimally invasive procedure under close perioperative monitoring. Close monitoring during and after the procedure revealed no significant bleeding complications. The patient recovered well, with follow-up at one year confirming therapeutic efficacy. This case illustrates that an individualized perioperative management strategy may mitigate bleeding risk in a patient with VWD undergoing transcatheter ASD closure.

As a single case report, this study has limitations regarding the generalizability of its conclusions. First, the successful management experience from this case may not be directly applicable to all VWD types or more complex cardiac surgery scenarios. Second, due to resource constraints, VWF multimer analysis was not performed, which is important for precise typing, long-term management, and genetic counseling.

## Conclusions

The diagnostic and management process in this case provides important insights. First, the APTT mixing study can be useful for differentiating the causes of coagulation abnormalities for differentiating the cause of coagulation abnormalities, and its results can guide subsequent precise diagnosis. For any patient scheduled for surgery, even in the absence of an obvious bleeding history, mild abnormalities in coagulation screening should be taken seriously and used as a starting point for further investigation. Second, when reduced FVIII activity is found, VWD should be included in the routine differential diagnosis. Finally, for surgical patients diagnosed with VWD, a multidisciplinary team (MDT) approach should be adopted to formulate a meticulous perioperative plan, thereby maximizing patient safety.

## Data Availability

The raw data supporting the conclusions of this article will be made available by the authors, without undue reservation.
